# Precision Medicine in Adult Obstructive Sleep Apnea and Home Diagnostic Testing: Caution in Interpretation of Home Studies Without Clinician Input Is Necessary

**DOI:** 10.3389/fneur.2022.825708

**Published:** 2022-02-21

**Authors:** Timothy Quy-Phong Do, Stuart Grayson MacKay, Matthew Eugene Lam, Anders William Sideris, Andrew Christopher Jones, Lyndon Sidney Chan

**Affiliations:** ^1^Illawarra ENT Head and Neck Clinic, Wollongong, NSW, Australia; ^2^Department of Otolaryngology, Head and Neck Surgery, Wollongong Hospital, Wollongong, NSW, Australia; ^3^Illawarra Health and Medical Research Institute, University of Wollongong, Wollongong, NSW, Australia; ^4^Department of Respiratory Medicine, Wollongong Hospital, Wollongong, NSW, Australia; ^5^Illawarra Sleep Medicine Centre, Wollongong, NSW, Australia

**Keywords:** home sleep study, polysomnography, sleep medicine, obstructive sleep apnea, otolaryngology

## Abstract

**Purpose:**

To assess the validity of home sleep apnea test directed diagnosis and treatment of obstructive sleep apnea (OSA) in a real-life clinical setting and establish the extent to which clinical evaluation alters diagnosis and therapeutic intervention, in the context of the evolving realm of precision medicine.

**Methods:**

Retrospective consecutive cohort study of 505 patients referred to a single center between 15th September 2015 to 14th September 2016, multidisciplinary specialist sleep clinic presenting with a home sleep apnea test prior to referral. We evaluated the effect of sleep medicine practitioner (SMP) and ear, nose, and throat surgeon (ENTS) review on patient diagnoses, disease severity, and management options in OSA.

**Results:**

Hundred and fifteen patients were included. Repeat evaluation with in-lab polysomnogram (PSG) was required in 46/115 (40.0%) of patients, of which 20/46 (43.5%) had OSA severity changed. Sleep medicine practitioner review decreased the need for repeat testing with formal in-lab PSG (*p* < 0.05) and increased patient acceptance of continuous positive airway pressure (CPAP) as a long-term management option for OSA. Sleep medicine practitioner/ENTS review resulted in discovery of a non-OSA related sleep disorder or change in OSA severity in 47.8% (55/115). Ear, nose, and throat surgeon review resulted in additional or changed diagnosis in 75.7% (87/115) of patients.

**Conclusion:**

In the clinical assessment and diagnosis of OSA, patients should be reviewed by medical practitioners with an interest in sleep disorders to better navigate the complexities of assessment, as well as the identification of co-morbid conditions.

## Introduction

The prevalence of adult obstructive sleep apnea (OSA) can vary significantly based on factors such as apnea-hyponea index (AHI), scoring criteria and test type (1). The prevalence in Australia is estimated to be 3.9%, leading to an estimated $943.8 million Australian dollars' worth of health care costs, as well as $13.4 billion in associated financial cost from productivity loss, work absenteeism, and reduced employment (2019–20) (2). Without appropriate treatment, the consequences of OSA include increased risks of cardiovascular disease (hypertension, heart failure, arrythmias), stroke, and diabetes (3). Untreated OSA may also lead to daytime somnolence, impaired concentration, and cognitive function and ultimately affect quality of life (QoL) (4, 5). Therefore, clinical correlation with polysomnographic findings is critical in defining risk and guiding treatment advice.

Sleep disorders can overlap, with OSA often co-existing with chronic insomnia, circadian misalignment, and other disorders. This adds further complexity to diagnosis, meaning that when OSA is suspected, a comprehensive sleep assessment is important to personalize subsequent diagnostic investigations and treatment.

Laboratory attended polysomnography (PSG) is a diagnostic investigation for sleep-related breathing disorders that collects the following variables—sleep stages (*via* electroencephalograhy, electrooculogram, submental electromyography), respiratory effort, airflow, snoring, end-tidal carbon dioxide, transcutaneous PCO_2_, oxygen saturations, electrocardiogram, body position, and limb movements. An in laboratory attended PSG is the gold standard and is defined as a type 1 study. This is the basis by which other tests are compared. Type 2 devices involve an unattended polysomnography where the same equipment as Type 1 is used, however a technician is not present during the study to troubleshoot. Type 3 studies involve portable devices and collect fewer physiological variables (typically four to seven). Portability of level 2 and 3 studies means that they can be performed out of the laboratory and are often referred to as home sleep apnea testing (HSAT). Type 4 evaluations involve portable devices with one to two variables. In recent years, with the emergence of mobile technology, developers have been engineering novel tools and mobile device sensors that utilize motion/actigraphy measurements, and audiological and video recording (6).

The decision between which diagnostic study is nuanced and must be tailored to the individual patient. Patients may request home testing due to convenience and increased accessibility (7). A 2018 study comparing patient satisfaction associated with in-lab PSG vs. HSAT, demonstrated increased satisfaction with in-lab PSG—suggesting the importance of other factors such as perceived competence of staff involved, perceived value, test results, and treatment outcomes (8). Therefore, when deciding between HSAT and PSG, the convenience and accessibility must be carefully weight against the potential harms that may result from misdiagnosis and inappropriate treatment or lack of treatment. Additionally, in-lab PSG testing may be required if home assessment is inadequate or inconclusive (9). The AASM 2017 guidelines (9) recommend that uncomplicated patients with signs and symptoms of moderate to severe OSA should undertake either PSG or HSAT. Patients with significant cardiorespiratory disease, suspected respiratory muscle weakness due to neuromuscular conditions, awake hypoventilation or suspected sleep related hypoventilation, chronic opioid medication, stroke, or history of severe insomnia should undergo PSG instead of HSAT due to the risk of inaccurate assessment and treatment (9).

Currently in Australia, HSAT is performed without clinical review by a qualified adult sleep medicine practitioner (SMP) and facilitates presentation to a sleep service without prior assessment of their sleep disorder (10). Sleep medicine practitioners are also responsible for the prescription and titration of CPAP in Australia. Ear nose and throat surgeons (ENTS) are commonly involved as part of the multidisciplinary management of patients with OSA and can facilitate nasal and upper-airway management including surgery. This paper aims to assess the validity of HSAT directed diagnosis and treatment in a real-life clinical setting and establishes the extent to which clinical evaluation alters diagnosis and therapeutic intervention, in the context of the evolving realm of precision medicine. This study also aims to provide a review of the updated literature around the decision-making paradigms when it comes to personalizing the diagnostic pathway.

## Methods

Ethics approval was obtained prior to conducting the study from the University of Wollongong/Illawarra Shoalhaven Local Health District Human Research Ethics Committee (2017/003). A retrospective cohort study within a combined tertiary clinic involving ENTS and SMP was performed. All patients referred and seen in this tertiary OSA clinic within a one-year period from 15th September 2015 to 14th September 2016 who had a pre-referral HSAT were included. Patients were excluded if they did not have a HSAT prior to review, or if a level 1 sleep study had already been performed.

Demographic data, sleep study data, SMP, and ENTS clinical review results, including Epworth Sleepiness Scale (ESS), pretest probability, diagnosis, and management were scrutinized. Ear, nose, and throat and SMP review occurred jointly and involved thorough history and examination including flexible nasendoscopy if required. Patients were considered somnolent if their ESS was >12, chosen to indicate moderate to severe sleepiness, and exclude mild/borderline ESS of 10–12 (11). Incongruence of sleepiness was determined if the patient ESS was >12 but had a HSAT diagnosis of no or mild OSA. Pretest probability of OSA obtained from medical record review, with high probability being based on the presence of 2 or more STOP BANG questionnaire positive responses in addition to either being male or having a body mass index ≥35 (12). Pretest probability incongruence was determined by having a high pretest probability but with a HSAT diagnosis of no or mild OSA. Patients with incomplete data were excluded. Polysomnography was performed based on clinical assessment if the initial test was incongruent with pre-test probability, inconclusive or technically inadequate (9). Patient acceptance of continuous positive airway pressure (CPAP) was based on history and interrogation of CPAP machine readings at the conclusion of data collection.

Categorical data requiring analysis was performed with SPSS (IBM Corp. Released 2013. IBM SPSS Statistics for Mac, Version 22.0. Armonk, NY, USA). Figures were created with Prism (GraphPad Software. Released 2020. Prism 9 for Windows 64-bit, Version 9.0.0. San Diego, CA, USA)

## Results

**Figure 1:**
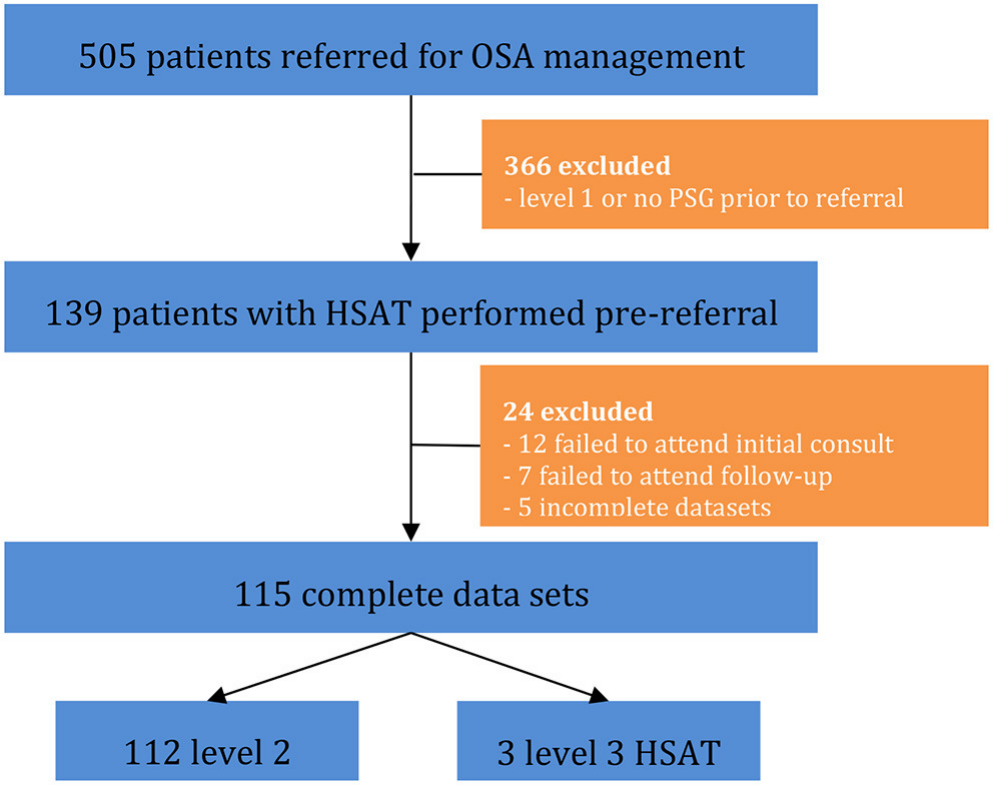
Flowchart of data collection process.

A total of 505 patients were referred for OSA management, with selection criteria and exclusions represented in Figure 1. Ultimately, 115 patients had level 2 or 3 studies (HSAT) performed and complete datasets for analysis.

Study population demographics and severity of OSA according to HSAT are shown in Table 1. Sleep medicine practitioner review prior to HSAT was performed in 10/115 (8.7%) patients. High pre-test probability of OSA was determined in 89/115 (77.4%) of patients. Level 2 HSAT was performed in 112/115 (97.4%) patients, with the remaining three patients undergoing level 3 studies.

**Table 1:** Baseline characteristics for demographics and HSAT results.

**Baseline characteristics**	**Number (range or %)**
Demographics	
Age, years	41.9 (21–70)
Gender, male	91 (79.1%)
BMI, kg/m^2^	29.1 (18.0–55.3)
HSAT outcomes	
AHI, events/h	26.3 (1–86)
No OSA (AHI <5)	12 (10.4%)
Mild OSA (AHI 5–14)	28 (24.3%)
Moderate OSA (AHI 15–30)	33 (28.7%)
Severe OSA (AHI >30)	42 (36.5%)

*Numbers are represented as mean with range or as absolute values with percentage*.

**Figure 2:**
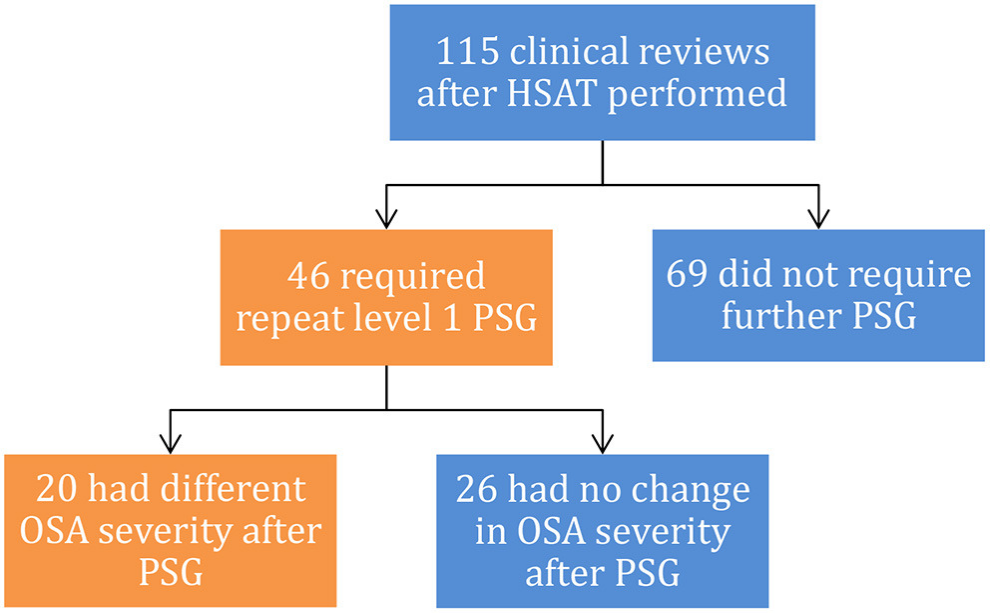
Summary of patients requiring repeat PSG after HSAT.

Study results are shown in Figure 2. Two of the level 3 studies were reported by a general practitioner (GP), all three level 3 studies had inappropriate use of the term AHI as defined by AASM (13).

**Figure 3:**
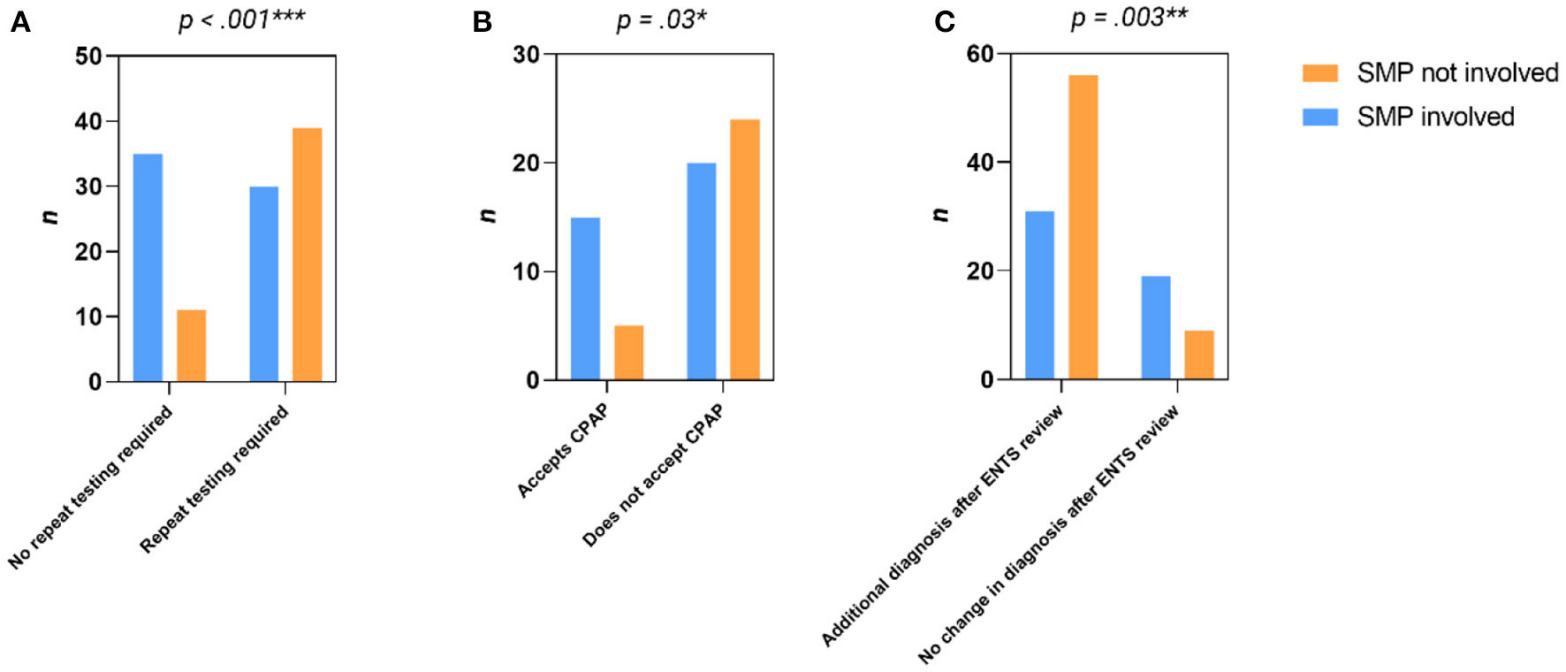
Contingency analysis of: **(A)** Impact of SMP review on repeat testing, **(B)** Impact of SMP review on acceptance of CPAP therapy and **(C)** Impact of SMP review on additional diagnosis after ENTS review.

Repeat evaluation with in-lab PSG was required in 46/115 (40.0%) of patients, of which 20/46 (43.5%) had OSA severity changed (Figure 2). Having a SMP review significantly decreased the need for repeat testing [22.0 vs. 53.9%, $X_{\left(1,\text{n}=115\right)}^{2}$ = 11.9, *p* < 0.001] with Chi-squared test (Figure 3A).

Ultimately 64 patients had a trial of CPAP, 29/64 (45.3%) without SMP supervision. Sleep medicine practitioner supervision significantly improved patient acceptance of CPAP as long-term management [17.3 vs. 42.9%, $X_{\left(1,\text{n}=64\right)}^{2}$ = 2.2, *p* = 0.03] with Chi-squared test (Figure 3B).

**Figure 4:**
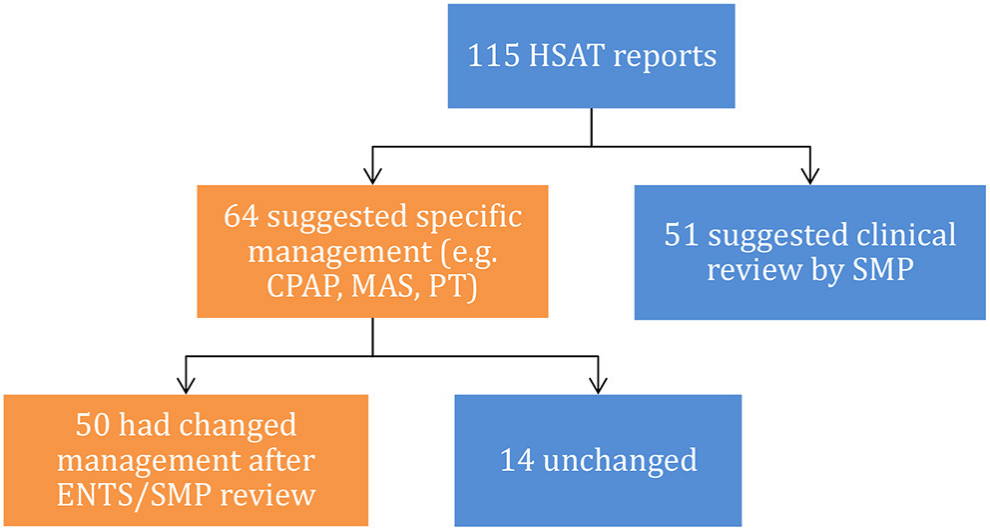
Subsequent management algorithm alterations based on HSAT recommendations.

ENTS and/or SMP review resulted in discovery of a non-OSA related sleep disorder or had the severity of OSA changed in 55/115 (47.8%) patients. Ear, nose, and throat surgeon review resulted in an additional or changed diagnosis in 87/115 (75.7%) of patients (see Table 2 for details). Prior SMP review significantly decreased this likelihood [62.0 vs. 86.2%, $X_{\left(1,\text{n}=115\right)}^{2}$ = 9.0, *p* = 0.003] on Chi-squared test (Figure 3C). 41/115 (29.5%) patients had more than one additional diagnosis. Clinical review by ENTS and/or SMP review changed the management plan in 50/64 (78.1%) HSAT reports that made treatment recommendations (Figure 4). Of the entire cohort, ENTS were able to provide treatment options in 91/115 (79.1%) patients with further details in Table 2.

**Table 2:** Diagnostic additions and treatment options offered due to ENTS review.

**Findings**	***n***	**Treatment**	***n***
Nasal obstruction	58	Upper airway reconstructive surgery nasal surgery + CPAP	23
Non-OSA related sleep disorder	15	Medical nasal management + CPAP	22
Elevated BMI	15	Mandibular advancement splint Weight loss	20
Non-nasal upper airway anatomy abnormality	13		16
Orthodontic issues		Positional therapy	15
Positional OSA	9	Sleep psychology	12
Maxillofacial abnormality	6	Maxillofacial/orthodontic review	11
Gastroesophageal reflux disease (GORD)	4	Nasal valve dilators + CPAP Management of GORD	4
Other ENT disorder	3	No management required	4
			3
	3		4

## Discussion

The diagnosis and management of adult OSA is complex and should integrate the patient's underlying complaint(s), anatomy, level/generators of dynamic airway collapse, physiology, and patient personal preferences. The process often benefits from a specialized multidisciplinary approach that aims to provide streamlined and individualized care (14, 15). In Australia, this process is guided by the GP, who can refer for a HSAT and treat the patient with referral to a specialist as required. This is a valid and important treatment path (16), and if effective, allows patients to have their sleep problem managed in the fastest and most cost effective way. However, this paper highlights the challenges that may exist when a one-size fits all algorithm is applied, rather than a personalized approach.

Of the patients who required repeat PSG, 43.5% had an adjusted severity of OSA. Within our data set, SMP review significantly decreased the requirement for subsequent PSG, demonstrating the importance of SMP review for accurate diagnosis and management in those failing treatment for presumed OSA. Sleep medicine practitioner and ENTS are likely more nuanced at discovering non-OSA and overlapping sleep disorders such as insomnia and sleep related movement disorders, as well as niche diagnoses such as positional OSA (17, 18). This was supported by the 75.7% additional diagnosis after review with ENTS in our cohort. Additionally, SMP also have more resources and expertise in managing these sleep disorders, with potential access to a multidisciplinary team, and ability to apply appropriate treatments, deal with initial treatment failures and monitor long term outcomes (14). Again, this highlights the importance of patient history and examination in the personalization of the diagnosis and management of OSA.

**Figure 5:**
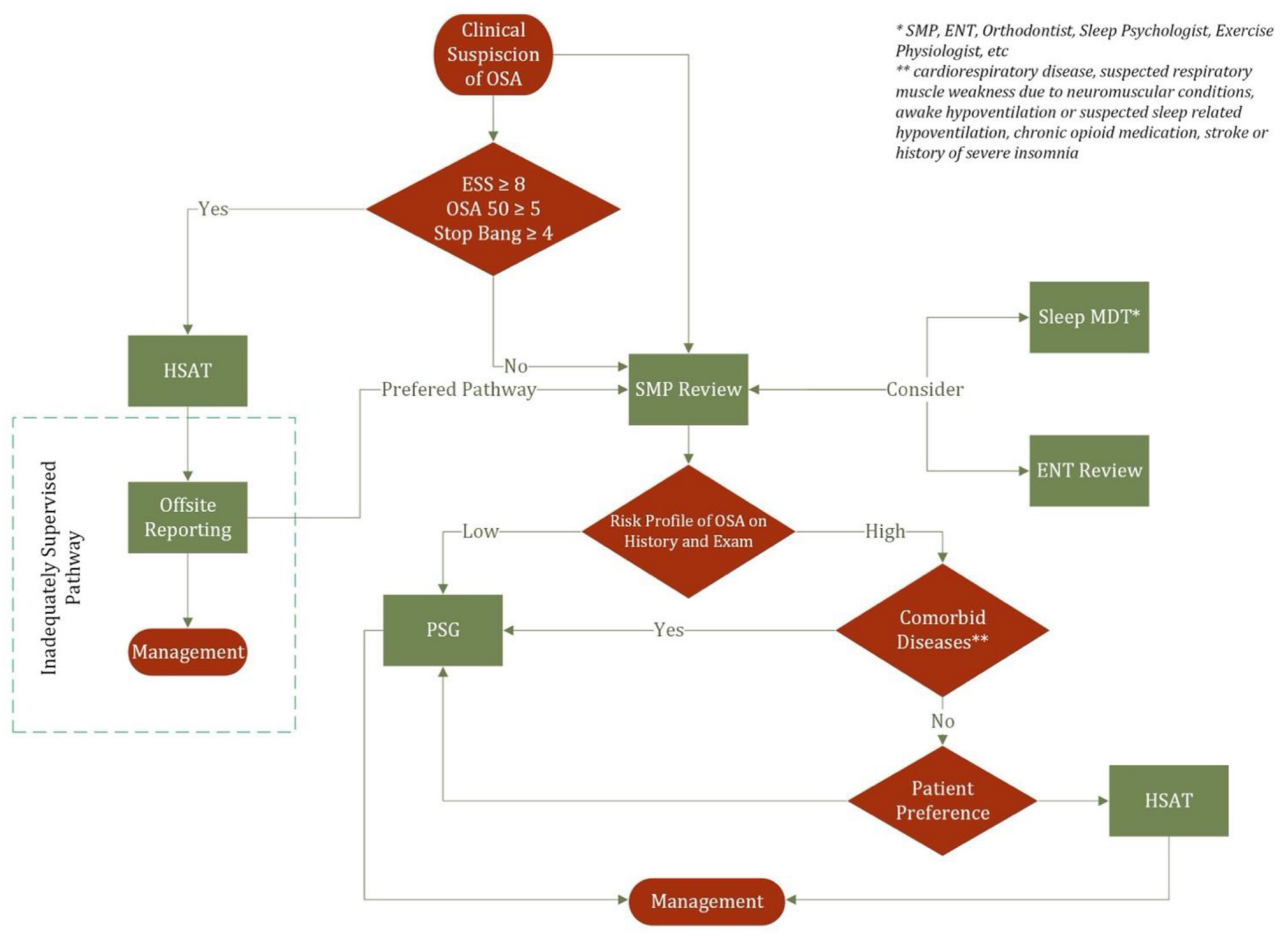
Decision making algorithm for sleep apnea testing.

The decision regarding type of sleep study also varies according to geography. In the Australian context, patients with a high probability of moderate to severe OSA could be offered types I, II, III, or IV sleep studies in the absence of significant medical comorbidities and other sleep related diseases (19). If a Type III or IV study is performed and is negative or non-diagnostic, then Type I or II should be performed. Furthermore, patients with low probability of moderate to severe disease could be offered type I and II. Such a paradigm for can be similarly found in the American Academy of Sleep Medicine guidelines (9) that suggest that in populations with “increased risk of moderate to severe OSA, both the increased likelihood of false negatives and significant impact of missed diagnosis” warrants the use of PSG as the subsequent test should HSAT is negative or non-diagnostic. European guidelines following a similar concept however differ in that low risk patients are recommended Type I studies and not Type II (20). Figure 5 demonstrates an algorithm for the decision making between PSG and HSAT which serves to highlight a current deficiency in the Australian practice where patients may be prescribed CPAP prior to SMP review. This paper underpins the importance of SMP review in the personalization of diagnostic investigation and offers a potential solution to the inadequately supervised OSA management pathway.

Ear, nose, and throat surgeon involvement with sleep disorders should be concentrated on management of OSA. Unless clearly indicated, as seen in anatomical nasal obstruction or presence of other non-sleep related otorhinolaryngology diagnosis, SMP review should ideally precede or be simultaneous with ENTS. However, ENTS treating patients with sleep disturbance need to recognize the presence of other sleep disorders and understand the role of surgery for OSA. Use of a surgical checklist (21) may help.

Our study demonstrates statistically and clinically significant increased compliance with CPAP after review that involves a SMP. The management of non-adherence with CPAP is another area in which a personalized approach is critical. Educational, behavioral, and troubleshooting interventions may be useful both before and during the initiation of CPAP to mitigate the chance of non-adherence (22, 23). Other strategies should be tailored to the individual's concerns, for example comfort, air leak, extent of humidification, and preference for nasal or oral mask. Patients with associated nasal obstruction from deviated septum or other pathology may benefit from facilitatory nasal surgery including septoplasty and/or turbinoplasty, which can reduce CPAP pressure requirements (24).

Clinicians must also be cognisant of the economic impact of the diagnostic test selected. Although care must be taken when considering these economic analyses (due to imprecise modeling and limited data) (9), HSAT management pathways are generally considered cost effective for the patient, but more costly for the healthcare provider (25). The decreased cost of HSAT must be weighed against the potential for false negative and false positive results and subsequent under-treatment and over-treatment of patients. Our data demonstrates ENTS and/or SMP review changed the management plan in 78.1% of patients that were recommended treatment with CPAP without SMP review. Whether this affects long term patient outcomes (including cardiometabolic and neurological outcomes, sleepiness, or QoL) is beyond the scope of this study. Meta-analysis of clinical outcomes between HSAT and PSG pathways have demonstrated that subjective sleepiness is similarly improved both, without a significant difference in QoL (9).

Novel devices and emerging technologies are increasingly used by patients as a screening tool for OSA with such technologies as ultrasound, smart watches, bed sensors, wireless electroencephalography, and radiofrequency sensors becoming increasingly prevalent (26). One meta-analysis of 18 studies demonstrated a 92% sensitivity in bed/mattress-based devices with the sensitivity decreasing and specificity increasing at higher AHI thresholds (6). Contactless smartphone technologies at best demonstrated a 97% sensitivity for mild OSA but had a high false positive rate of 0.49.

Our study has several limitations. It is a descriptive retrospective cohort of a single institution; hence future studies should draw conclusions from multiple site and referral sources. Given the component of subjectivity in OSA management, diagnosing additional disorders, HSAT interpretation, and threshold for CPAP compliance unintended biases may be introduced into our results. Assessment, treatment, and referral pathways may differ according to location and service availability. The Australian medical system may have points of difference from those around the world.

Rapid progression toward use of home testing and newer technologies may open the doorway to manipulation (especially with hypopnoea), over-scoring, and even over-treatment, particularly if such intervention bias leads to financial or other gain (27). Caution in real life practice is required, in order not to dispose of clinical assessment and correlation with diagnostics by well-trained SMPs and ENTS, as the adult OSA field moves toward precision medicine.

This paper highlights the potential challenges that exist in diagnostic algorithms for patients with adult OSA, particularly when considering the importance of a personalized approach to medicine. In an Australian context, the role of SMP and ENTS in the diagnosis and management of OSA should not be overlooked, as co-morbid sleep and airway conditions are often identified.

Despite the rapid advancement in novel and mobile home sleep technologies, this study demonstrates why we must remain cognisant of the potential challenges faced in the diagnosis and management of adult OSA.

## Data Availability Statement

The original contributions presented in the study are included in the article, further inquiries can be directed to the corresponding author.

## Ethics Statement

The studies involving human participants were reviewed and approved by University of Wollongong/Illawarra Shoalhaven Local Health District Human Research Ethics Committee (2017/003). Written informed consent for participation was not required for this study in accordance with the national legislation and the institutional requirements.

## Author Contributions

TD and ML: production of manuscript and data analysis. SM and AJ: production of manuscript, study conceptualization, and clinical management of patients. AS: production of manuscript. LC: production of manuscript, data processing, data analysis, and study conceptualization. All authors contributed to the article and approved the submitted version.

## Conflict of Interest

The authors declare that the research was conducted in the absence of any commercial or financial relationships that could be construed as a potential conflict of interest.

## Publisher's Note

All claims expressed in this article are solely those of the authors and do not necessarily represent those of their affiliated organizations, or those of the publisher, the editors and the reviewers. Any product that may be evaluated in this article, or claim that may be made by its manufacturer, is not guaranteed or endorsed by the publisher.
